# Ablation of lncRNA *Miat* attenuates pathological hypertrophy and heart failure

**DOI:** 10.7150/thno.50990

**Published:** 2021-07-06

**Authors:** Liu Yang, Jianxin Deng, Wenxia Ma, Aijun Qiao, Shiyue Xu, Yang Yu, Chan Boriboun, Xiang Kang, Dunzheng Han, Patrick Ernst, Lufang Zhou, Jiawei Shi, Eric Zhang, Tao-Sheng Li, Hongyu Qiu, Shinichi Nakagawa, Seth Blackshaw, Jianyi Zhang, Gangjian Qin

**Affiliations:** 1 Molecular Cardiology Program, Department of Biomedical Engineering, University of Alabama at Birmingham, School of Medicine and School of Engineering, Birmingham, AL 35294, USA.; 2 Feinberg Cardiovascular Research Institute, Northwestern University Feinberg School of Medicine, Chicago, IL 60611, USA.; 3 Department of Cardiology, Union Hospital, Tongji Medical College, Huazhong University of Science and Technology, Wuhan, China.; 4 Department of Medicine, University of Alabama at Birmingham, Birmingham, AL 35294, USA; 5 Department of Stem Cell Biology, Atomic Bomb Disease Institute, Nagasaki University, Nagasaki 852-8523, Japan.; 6 Center for Molecular and Translational Medicine, Institute of Biomedical Science, Georgia State University, Atlanta, GA 30303, USA.; 7 Faculty of Pharmaceutical Sciences, Hokkaido University, Sapporo 060-0812, Japan.; 8 Solomon H. Snyder Department of Neuroscience, Johns Hopkins University School of Medicine, Baltimore, MD 21205, USA.

**Keywords:** lncRNA, *Miat*, cardiac hypertrophy, heart failure, RNA splicing, cardiomyocytes

## Abstract

**Rationale:** The conserved long non-coding RNA (lncRNA) myocardial infarction associate transcript (*Miat*) was identified for its multiple single-nucleotide polymorphisms that are strongly associated with susceptibility to MI, but its role in cardiovascular biology remains elusive. Here we investigated whether *Miat* regulates cardiac response to pathological hypertrophic stimuli.

**Methods:** Both an angiotensin II (Ang II) infusion model and a transverse aortic constriction (TAC) model were used in adult WT and *Miat*-null knockout (*Miat*-KO) mice to induce pathological cardiac hypertrophy. Heart structure and function were evaluated by echocardiography and histological assessments. Gene expression in the heart was evaluated by RNA sequencing (RNA-seq), quantitative real-time RT-PCR (qRT-PCR), and Western blotting. Primary WT and *Miat*-KO mouse cardiomyocytes were isolated and used in Ca^2+^ transient and contractility measurements.

**Results:** Continuous Ang II infusion for 4 weeks induced concentric hypertrophy in WT mice, but to a lesser extent in *Miat*-KO mice. Surgical TAC for 6 weeks resulted in decreased systolic function and heart failure in WT mice but not in *Miat*-KO mice. In both models, *Miat*-KO mice displayed reduced heart-weight to tibia-length ratio, cardiomyocyte cross-sectional area, cardiomyocyte apoptosis, and cardiac interstitial fibrosis and a better-preserved capillary density, as compared to WT mice. In addition, Ang II treatment led to significantly reduced mRNA and protein expression of the Ca^2+^ cycling genes Sarcoplasmic/endoplasmic reticulum Ca^2+^ ATPase 2a (SERCA2a) and ryanodine receptor 2 (RyR2) and a dramatic increase in global RNA splicing events in the left ventricle (LV) of WT mice, and these changes were largely blunted in *Miat*-KO mice. Consistently, cardiomyocytes isolated from *Miat*-KO mice demonstrated more efficient Ca^2+^ cycling and greater contractility.

**Conclusions:** Ablation of *Miat* attenuates pathological hypertrophy and heart failure, in part, by enhancing cardiomyocyte contractility.

## Introduction

Cardiovascular disease is the leading cause of death worldwide 1. Under biomechanical stress such as hemodynamic overload or high levels of neurohormonal mediators, cardiomyocytes undergo hypertrophic growth to meet the environmental demands. However, sustained pathological cardiac hypertrophy can lead to heart failure 2, and the process is accompanied by a complex spectrum of pathophysiological changes, including cardiomyocyte Ca^2+^ mishandling, hypertrophic growth and death, vascular insufficiency, and interstitial fibrosis 3. Currently there is no specific treatment to effectively reverse cardiac pathological hypertrophy and reduce the morbidity and mortality of heart failure. Thus, uncovering new molecular mechanisms of cardiac remodeling has the potential to identify novel therapeutic targets 4.

Heart pump function is governed by cellular Ca^2+^ and excitation-contraction coupling 5. Upon pathological hypertrophic stimuli (e.g., pressure overload), cardiomyocytes increase contractility by augmenting the release of Ca^2+^ from the sarcoplasmic reticulum (SR), the major intracellular Ca^2+^ store, into the cytoplasm through RyRs (at systole) as well as the uptake of Ca^2+^ back into SR through SERCA2a (at diastole) 6. When the demand is not met, the cardiomyocytes compensate by synthesizing excess contractile proteins (hypertrophy), which jeopardizes the structural balance between subcellular organelles for efficient Ca^2+^ signaling, contraction/relaxation, and energy metabolism, and causes insufficiency of vascular supply. These changes collectively contribute to cardiomyocyte cell death including apoptosis, which in turn triggers a pro-inflammatory response, fibrosis, and abnormal accumulation of extracellular matrix proteins (e.g., collagens); excessive accumulation of collagen stiffens the ventricles, which further impairs contraction and relaxation and reduces capillary density. This adverse structural remodeling of ventricular walls increases oxygen diffusion distances, leading to myocardial ischemia that contributes to the transition from hypertrophy to failure 7. It is well recognized that impaired RyR2 function (e.g., Ca^2+^ leak) or decreased SERCA2a expression/activity can lead to reduced pump function and serial compensatory responses, including chronic activation of neurohormonal pathways and hypertrophic gene programming, which ultimately results in decompensation and heart failure 8. Thus, restorations of the activity and integrity of SERCA2a and RyR2 are promising therapeutic strategies 9, 10.

LncRNAs are an emerging class of regulatory molecules with no or limited protein-coding capacity 11. At present, only a small proportion of lncRNAs have been functionally validated in cardiovascular biology, including heart development 12, 13 and hypertrophy 14-16. *Miat*, also known as Retinal Non-Coding RNA 2 (RNCR2) or Gomafu, is an intergenic lncRNA highly conserved across species including mouse and human 17, 18. It is ~9 kb long, accumulated within the nucleus, and expressed in the central nervous system, heart, lung, and spleen 19, 20. In a large-scale genome-wide association study, *Miat* was identified to contain multiple single-nucleotide-polymorphisms (SNPs) in 6 loci that are strongly associated with increased morbidity of MI 19. Subsequently *Miat* has been suggested as a potential biomarker for left ventricular dysfunction in patients with MI and type 2 diabetes 21. Experimental evidence in rodents suggest that *Miat* promotes post-MI fibrosis 22 and cardiac hypertrophy 23-25 and contributes to diabetic microvascular dysfunction 26. However, due to the lack of a genetic *Miat* model, the function of *Miat* in cardiovascular disease has not been clearly defined.

In this study we examined the role of *Miat* in cardiac hypertrophy in newly-generated *Miat* KO mice. We found that KO of *Miat* protects hearts from Ang II-induced pathological hypertrophy and TAC-induced heart failure, and that this beneficial effect is at least partially attributable to the enhanced calcium handling and contractility of cardiomyocytes.

## Materials and Methods

### Mice

All the animal studies and operation procedures in this report were approved by the Institutional Animal Care and Use Committee (IACUC) of the University of Alabama at Birmingham and performed in compliance with the ''Guide for the Care and Use of Laboratory Animals'' (NIH publication) and all relevant ethical regulations for animal testing and research. The *Miat* KO mice were generated by deleting the entire *Miat* gene, including the two splicing variants, plus 70 bp upstream and 1 bp downstream sequences and genotyped as we previously described 20. Male, 8-10 week-old homozygote *Miat*-KO mice and their WT littermates on C57BL/6 background were used for all the experiments unless specified. Sample sizes were predetermined by power calculation with 80% power.

### Ang II infusion model

WT and *Miat*-KO mice received Ang II (2 mg/kg BW/day, Sigma Aldrich) or saline (control) continuously via Alzet mini-osmotic pumps (model 2004; Durect Corporation) for up to 28 days. Pumps were prepared and subcutaneously implanted in the mice by following the manufacturer's instructions.

### TAC model

TAC model was performed using a clip method 27. Briefly, mice were anesthetized by continuous inhalation of 2% isoflurane. A skin incision was made at the level of the suprasternal notch, followed by a longitudinal cut of ~3 mm in length in the proximal portion of the sternum. A metal clip calibrated to a 29-gauge needle was placed in the aortic arch between the innominate artery and the left common carotid artery. For the Sham group, the procedure was identical except the placement of the clip to induce constriction. Post-operative care was performed by following our approved animal study protocol. For pain management, Buprenex (0.05-0.2 mg/kg) was subcutaneously injected every 12 h for 48 h. In addition, Metacam (1 mg/kg) was subcutaneously injected at the end of surgery and continued daily for 3 d. Mice were euthanized 6 weeks later, and the tissues were isolated for histological and biochemical analysis.

### Echocardiography

Cardiac function was recorded and analyzed before and after Ang II infusion or TAC surgery, using a Vevo 2100 high-resolution ultrasound system (VisualSonics Inc) as we previously described 28. For systolic function, mice were conscious and secured with surgical tapes to the platform in supine position. Data were collected after three days of training. Parasternal long-axis view, short-axis view at the papillary muscle level and 2-D guided M-mode images were recorded. Left ventricular fractional shortening (FS), ejection fraction (EF), stroke volume (SV) and cardiac output (CO) were calculated. For diastolic function, mice were lightly anesthetized via 2% isoflurane inhalation, and heart rates were maintained at 350-450 beats per minutes. The transmitral left ventricular outflow tract Doppler spectra (E, A) was recorded from an apical four chamber view. Tissue Doppler was used to measure the lateral E' spectra, and to calculate the E/E' ratio. Measurements were performed by an individual who was blinded to the treatment assignments.

### Blood pressure (BP) measurement

Systolic and diastolic arterial BP in WT and *Miat*-KO mice were measured by a noninvasive tail-cuff BP analyzer (CODA System, Kent Scientific) as we described previously 29. Mice received 5 days of training before data collection. For each mouse, twenty measurements were collected and averaged.

### Histology

Both formalin-fixed paraffin-embedded (FFPE) and fixed frozen (FF) sections were used in this study. For FFPE sections, cardiac tissues were fixed in 10% formalin overnight and embedded in paraffin, as described previously 30. For FF sections, tissues were fixed in 4% paraformaldehyde for 4 h, dehydrated in 30% sucrose and embedded in OCT compound followed by cryosectioning. Three sections per heart and 6 fields per section were examined. The HE staining (ThermoFisher Scientific) was performed by following standard procedures. The Sirius Red/Fast Green (Chondrex), WGA (ThermoFisher Scientific) and TUNEL (Sigma Aldrich) staining were performed according to manufacturer's instructions. Anti-CD31 (Abcam, 1:50), Anti-cardiac troponin I (Abcam, 1:50) antibodies were used in the fluorescent immunohistology. All histological assessment and analyses were performed by an individual blinded to treatment assignments.

### Isolation of neonatal mouse cardiomyocytes

Cardiomyocytes were isolated from neonatal WT or *Miat*-KO mice after euthanasia. Briefly, the beating hearts were excised and transferred into ice-cold HEPES-buffered saline solution (Mg^2+^ and Ca^2+^ free). After removing the atria and large vessels, hearts were washed and minced into small pieces less than or equal to 1 mm^3^. Tissues were then digested 5 times by incubating at 37 °C in HEPES-buffered saline solution containing collagenase type II. After each digestion step, the supernatant was filtered (100 micron) and collected in DMEM/F12 supplemented with 20% FBS, 100 U/mL penicillin, and 100 μg/mL streptomycin. The cells were then centrifuged, resuspended and plated into T25 tissue-culture flasks at 37 °C. After 4 h, the non-adhesive cardiomyocytes were re-plated and cultured in DMEM/F12 supplemented with 10% FBS, 100 U/mL penicillin, 100 μg/mL streptomycin, and 0.1 mM bromodeoxyuridine.

### Lentiviral *Miat*-shRNA vector and *Miat* overexpression plasmid

Viral vectors encoding *Miat* short hairpin RNA (shRNA) were produced by co-transfection of 293FT cells with plasmid coding for *Miat* shRNA (pLKO.1-shRNA*^Miat^*-puro) or control scrambled shRNA (pLKO.1-shRNA^Scrambled^-puro), pMD2.G and psPAX2, as previously described 31. Medium was collected 48-72 h after transfection and concentrated by ultracentrifugation (2 h, 4 °C at 2,5000 rpm). Then the concentrated virus was carefully resuspended in PBS. For infection, the virus was suspended in fresh medium containing 8 µg/mL polybrene and applied onto HL-1 cells with a multiplicity of infection at 2 to 1. Three days later, the transduced cells were selected in 5 ug/mL puromycin and continued for 1 week. The shRNA sequences are: *Miat* shRNA: 5'-CCG GCC TAG AAA CCT GAT GTA GAC TCG AGT CTA CAT CAG GTT TCT AGG TTT TTG-3'; Control Scrambled shRNA: 5'-CCG GCC TAA GGT TAA GTC GCC CTC GCT CGA GCG AGG GCG ACT TAA CCT TAG GTT TTT G-3'. For *Miat* overexpression, plasmid pCAG-*Miat*-GFP coding for mouse *Miat* 17 or control plasmid pCAG-GFP was transfected into HL-1 cells using Amaxa Nucleofector (Lonza), following the manufacturer's instructions. The level of *Miat* expression was confirmed by qRT-PCR.

### Immunocytochemistry

Cells were cultured on chamber slides and fixed with cold 4% paraformaldehyde, then stained with primary antibodies specific for cardiac Troponin T (Abcam,1:100). Pertinent secondary antibodies were conjugated with Alexa Fluor 594. Nuclei were counterstained with DAPI (Vector Laboratories, Burlingame, CA). Sections were examined under a Nikon confocal microscope.

### Quantitative real-time RT-PCR (qRT-PCR)

qRT-PCR was performed as we described previously 32. Briefly, total RNA was extracted from heart tissues using the E.N.Z.A Total RNA Kit (Omega) and reverse transcribed into cDNA using PrimeScript RT Reagent Kit (TAKARA). The values were normalized to the levels of 18S. The sequences of all primers are reported in **Table S1**.

### Western Blotting

Western Blotting was performed via standard techniques as we described previously 32, by using antibodies against RyR (Abcam, Cambridge, UK), Serca 2a (Abcam, Cambridge, UK), and GAPDH (Cell Signaling Technology, Danvers, MA, USA).

### RNA sequencing (RNA-seq) and bioinformatics analyses

Left ventricular tissues were collected from WT or *Miat*-KO mice that received Ang II or saline treatment for 7 days. Total RNA was extracted using TRIzol (ThermoFisher Scientific). RNA samples were then sequenced as described elsewhere 33. Briefly, quality control testing was performed on all the RNA samples using the Agilent 2100 Bioanalyzer, followed by polyadenylate positive (poly A^+^) selection and conversion to cDNA. After library construction, paired-end sequencing runs were performed on the Illumina HiSeq 2500. All RNA-seq fastq Reads were aligned to the reference genome mouse mm10. Genes with a fold change greater than 2 (regardless of isoforms) and p<0.05 were used for Gene Ontology (GO) analyses (http://david.abcc.ncifcrf.gov/). Gene isoforms were analyzed by MISO (https://miso.readthedocs.io/en/fastmiso/) and MATS (http://rnaseq-mats.sourceforge.net/).

### Isolation of adult mouse cardiomyocytes

Adult mouse left ventricular cardiomyocytes were isolated as described 34. Briefly, hearts were excised from the heparinized and anesthetized mice, cannulated via the aorta and connected to a Langendorff perfusion system (Radnoti). Hearts were perfused for about 5 min with calcium-free perfusion solution, followed by digestion buffer containing 1 mg/mL type II collagenase (Worthington) for about 15 min. Both atria and right ventricle were trimmed off from the heart. Cells were then dissociated from the left ventricle to form a single cell suspension, followed by calcium reintroduction.

### Fractional shortening (FS) and intracellular calcium measurements

Immediately after isolation, cardiomyocytes were used to measure FS and intracellular calcium transients as we previously reported 35. Briefly, cells were loaded with 5 µM Fluo-4 AM (ThermoFisher Scientific, F14201, dissolved in DMSO containing 20% pluronic F-127) for 10 min at 37 °C and then perfused with Tyrode's solution (137 mM NaCl, 1.2 mM MgCl_2_, 1.2 mM NaH_2_PO_4_, 20 mM HEPES, 5.4 mM KCl, 1.8 mM CaCl_2_, 10 mM glucose, pH 7.4) in a heated chamber (37 °C) on the stage of an inverted fluorescent microscope (Olympus, Fv1000) to remove the dye and complete de-esterification. After the cardiomyocytes reached a steady state with field stimulations (1 Hz), line-scan images were acquired along the longitudinal axis of the cells. The F0 was measured right before stimulation and the maximal Fluo-4 fluorescence (F) was measured at peak amplitude. Calcium amplitude (∆F/F0, ∆F=F-F0) was calculated. FS of cardiomyocytes was measured with edge detection.

### Statistics

All values are reported as mean ± standard error of the mean (SEM). Unpaired two-tailed Student's *t* test was used to compare means of two groups. One-way or two-way analysis of variance (ANOVA) were used in multiple (> 2) group comparisons with one or two independent variables, respectively. p < 0.05 was considered statistically significant.

## Results

### Deletion of *Miat* attenuates concentric cardiac hypertrophy

**Figure 1:**
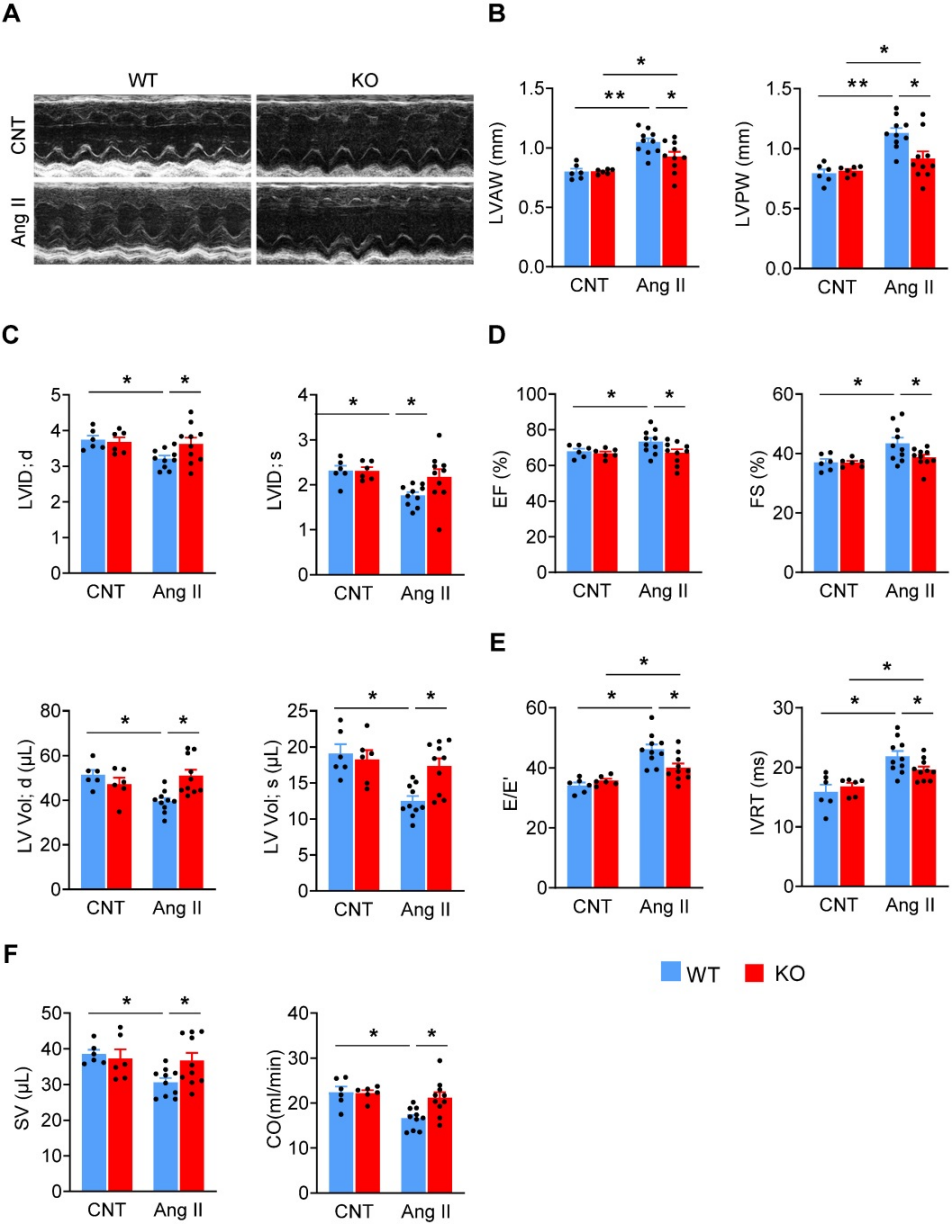
** Deletion of *Miat* attenuates Ang II-induced cardiac dysfunction.** *Miat*-KO mice and WT littermates were treated with Ang II (2 mg/kg BW/day) or saline control (CNT) via a subcutaneously-implanted mini-osmotic pump for 28 days, then cardiac functions were evaluated by echocardiography. (**A**) Representative image of echocardiography. (**B**) Thicknesses of LV anterior wall (LVAW) and posterior wall (LVPW). (**C**) LV internal diameter (LVID; *upper* panels) and volume (LV Vol; *lower* panels) at diastole (d) and systole (s). (**D**) LV ejection fraction (EF) and fractional shortening (FS). (**E**) Ratio between early mitral inflow velocity and mitral annular early diastolic velocity (E/E'; *left* panel) and isovolumic relaxation time (IVRT; *right* panel). (**F**) stroke volume (SV;* left* panel) and cardiac output (CO;* right* panel). *p < 0.05, **p < 0.01.

We firstly treated mice with Ang II (2 mg/kg/d) via a subcutaneously-implanted mini-osmotic pump, and at various time points analyzed *Miat* expression in the left ventricle (LV). *Miat* was significantly increased (~3 fold) at Day 7, then declined to baseline at Day 14 and 28 (**Figure S1**). To determine the role of *Miat* in cardiac hypertrophy, we used *Miat*-KO mice in which the entire *Miat* gene plus 71 bp of surrounding sequences have been removed (**Figure S2A-B**) 22. These KO mice appear developmentally normal and show no difference in gross appearance, body weight, or histological characteristics of major organs including the heart, as compared to WT littermates (**Figure S2C-F**). Ang II treatments dramatically increased the systolic and diastolic blood pressure in both WT and KO mice to similar levels (**Figure S3**). Notably, while Ang II induced a significant increase in the LV wall thickness (**Figure 1A-B, Table S2**) and decrease in the LV internal diameter and volume (**Figure 1C**) in WT mice, these structural changes were significantly attenuated in KO mice, indicating that *Miat*-deletion reduces Ang II-induced concentric hypertrophy. Interestingly, the attenuated hypertrophy was not observed until Day 21 of Ang II infusion in *Miat*-KO mice (**Figure S4**). Although WT mice displayed a slight increase in their ejection fraction (EF) and fractional shortening (FS) (**Figure 1D**), their diastolic function was markedly impaired after Ang II treatment, as indicated by increased E/E' and isovolumic relaxation time (IVRT) (**Figure 1E**). The overall cardiac function of WT mice was significantly reduced as indicated by the reduced stroke volume and cardiac output (**Figure 1F**); in contrast, these functional impairments were markedly attenuated in KO mice.

**Figure 2:**
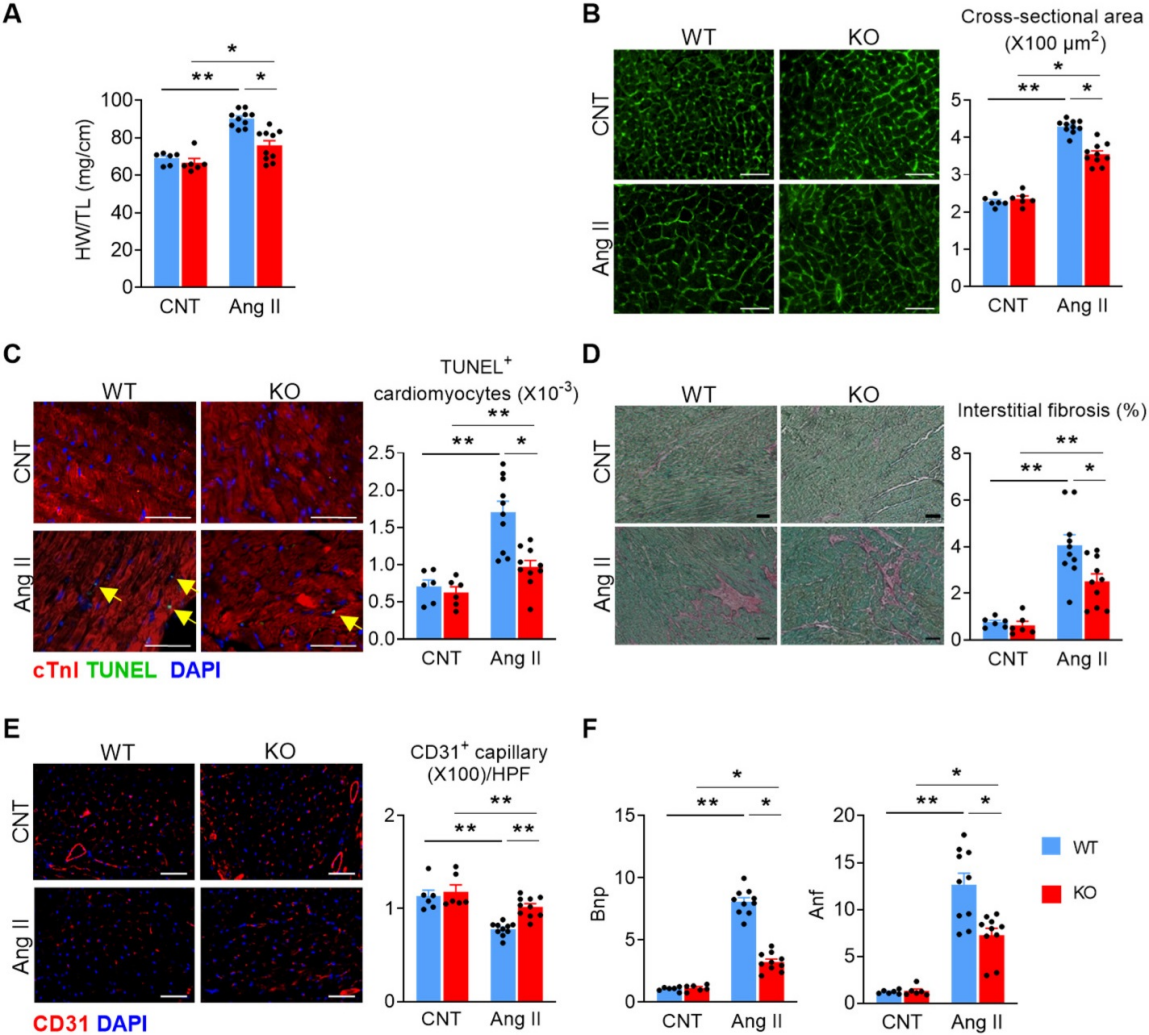
** Deletion of *Miat* attenuates Ang II-induced cardiac pathological hypertrophy.** At 28 days after continuous Ang II (2 mg/Kg BW/day) or saline treatment, mice were euthanized and examined. (**A**) Heart weight (HW) versus tibia length (TL) ratio. (**B**) Representative images of wheat germ agglutinin (WGA) staining (*left* panel) and quantification of cardiomyocyte cross-section areas (*right* panel). (**C**) Representative images of TUNEL and cTnI staining (*left* panel) and quantification of apoptotic cardiomyocytes (*right* panel*,* % of nuclei). Yellow arrows indicate TUNEL^+^ cardiomyocytes. Scale bar = 50µm. (**D**) Representative images of Sirius Red/Fast Green staining (*left* panel) and quantification of interstitial fibrosis areas (*right panel*). (**E**) Representative images of CD31 staining (*left* panel) and quantification of capillary density (*right* panel). HPF, high-powered field. (**F**) qRT-PCR analyses of the mRNA levels of Bnp and Anf in the heart tissue. *p < 0.05, **p < 0.01.

We then dissected the hearts from WT and KO mice to evaluate cardiac remodeling histologically. Ang II treatment significantly increased heart-weight to tibia-length ratio (**Figure 2A**) and cardiomyocyte cross-sectional area (**Figure 2B**), which was attenuated in KO mice. KO mice displayed significantly reduced cardiomyocyte apoptosis (**Figure 2C**), interstitial fibrosis (**Figure 2D**), and a better-preserved capillary density after Ang II treatment (**Figure 2E**). Furthermore, the mRNA levels of brain natriuretic peptide (Bnp) and atrial natriuretic factor (Anf) were significantly increased in the heart tissue of both WT and KO mice, but to a lesser extent in KO mice (**Figure 2F**). These data indicate that *Miat* deletion or inhibition alleviates Ang II-induced adverse cardiac remodeling. Furthermore, deletion of *Miat* attenuated Ang II-induced hypertrophy of primary cardiomyocytes *in vitro* (**Figure S5**), and knockdown (KD) of *Miat* in the HL-1 cardiomyocyte cell line diminished Ang II-induced expression of Bnp and Anf. Notably, overexpression (OE) of *Miat* in HL-1 cells increased, while restoration of *Miat* expression in* Miat*-KD HL-1 cells partially rescued, Ang II-induced expression of the cardiac hypertrophy genes (**Figure S6 & Figure S7**). Thus, *Miat* promotes cardiomyocyte hypertrophic growth autonomously.

### Deletion of *Miat* attenuates eccentric cardiac hypertrophy and heart failure

**Figure 3:**
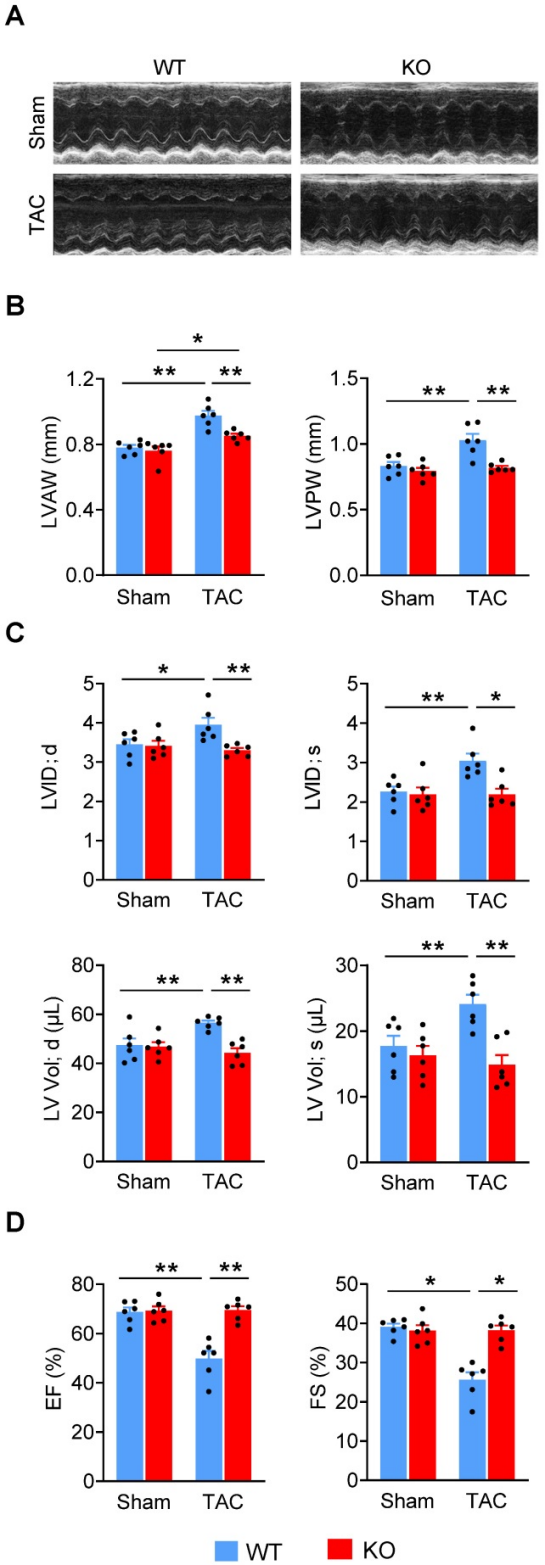
** Deletion of *Miat* attenuates TAC-induced heart failure.** Cardiac functions were evaluated with echocardiography in *Miat*-KO mice and WT littermates 6 weeks after TAC or Sham surgery. (**A**) Representative image of echocardiography. (**B**) Thicknesses of LV anterior wall (LVAW) and posterior wall (LVPW). (**C**) LV internal diameter (LVID; *upper* panels) and volume (LV Vol; *lower* panels) at diastole (d) and systole (s). (**D**) LV ejection fraction (EF) and fractional shortening (FS). *p < 0.05, **p < 0.01.

**Figure 4:**
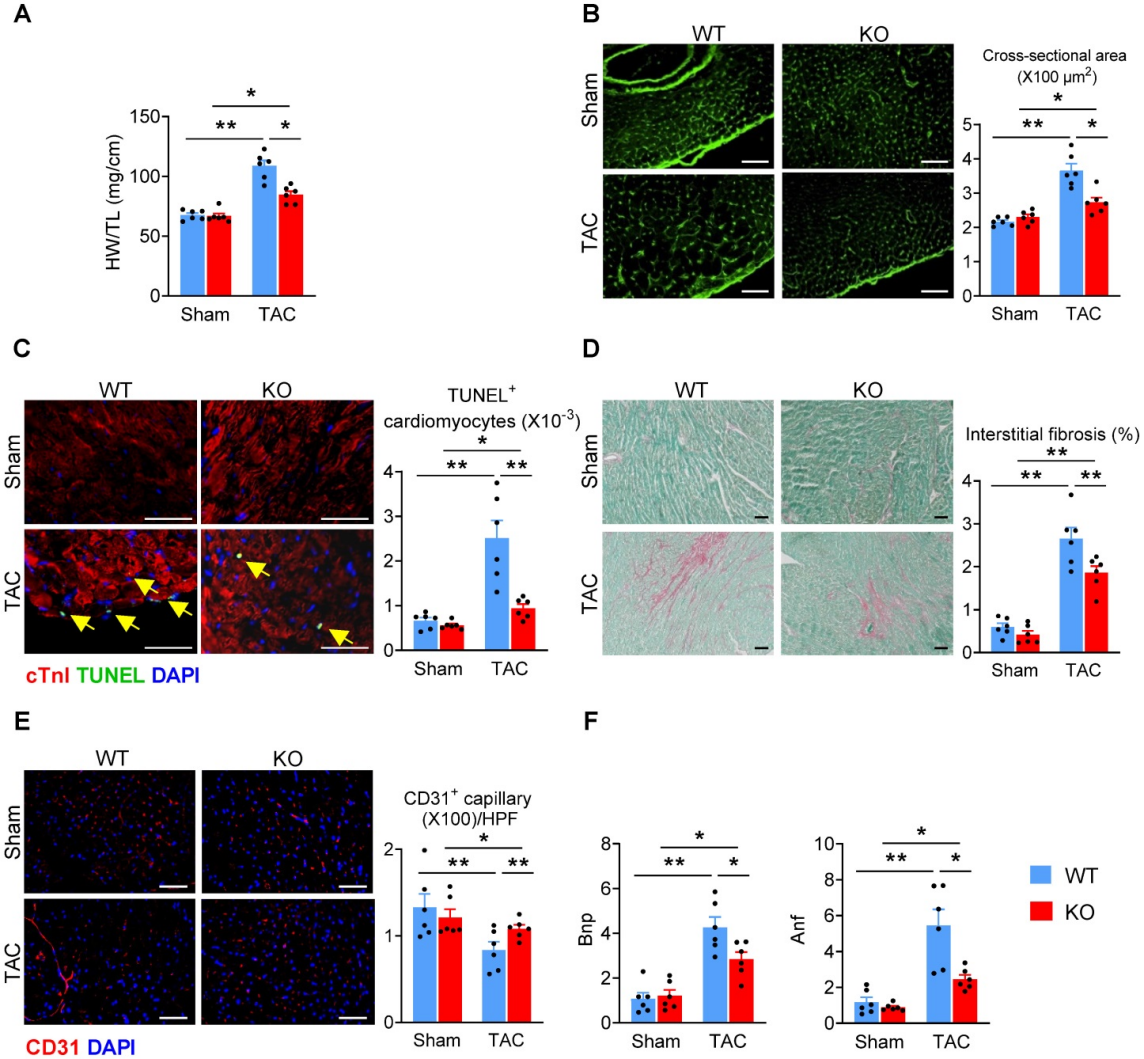
** Deletion of *Miat* attenuates TAC-induced cardiac adverse remodeling.** At 6 weeks after TAC or Sham surgery, mice were euthanized and examined. (**A**) Heart-weight (HW) to tibia-length (TL) ratio. (**B**) Representative images of WGA staining (*left* panel) and quantification of cardiomyocyte cross-section areas (*right* panel). (**C**) Representative images of TUNEL and cTnI staining (*left* panel) and quantification of apoptotic cardiomyocytes (*right* panel, % of nuclei). Yellow arrows indicate TUNEL^+^ cardiomyocytes. Scale bar = 50 µm. (**D**) Representative images of Sirius Red/Fast Green staining (*left* panel) and quantification of interstitial fibrosis areas (*right* panel). (**E**) Representative images of CD31 staining (*left* panel) and quantification of capillary density (*right* panel). HPF, high-powered field. (**F**) qRT-PCR analyses of the mRNA levels of Bnp and Anf in the heart tissue. *p < 0.05, **p < 0.01.

Since adverse cardiac remodeling and hypertrophy leads to heart failure, we examined the effects of *Miat* in heart failure by using the TAC model in *Miat*-KO and WT mice. Six weeks after surgery, although the LV wall was still thicker than baseline (**Figure 3A-B, Table S3**), the heart chamber (**Figure 3C**) was markedly dilated in WT mice, but not in *Miat*-KO mice. These structural changes in WT mice are consistent with a significant impairment in systolic cardiac function, as evidenced by a reduction in EF and FS (**Figure 3D**). KO mice displayed much better cardiac performance than the WT group (**Figure 3A-D**). In the gross and histological examinations, both heart-weight to tibia-length ratio and cardiomyocyte cross-sectional area were dramatically increased in WT mice, but to a lesser extent in KO mice (**Figure 4A-B**). Consistently, *Miat* deficiency results in reduced cardiomyocyte apoptosis (**Figure 4C**), interstitial fibrosis (**Figure 4D**), and a better-preserved capillary density (**Figure 4E**). The levels of Bnp and Anf were also significantly lower in KO mice (**Figure 4F**). Collectively, these data suggest that *Miat* deletion protects the heart from pressure overload-induced pathological hypertrophy and heart failure.

### Deletion of *Miat* blunts Ang II-induced cardiac hypertrophic gene expression

To gain molecular insights into the regulation of cardiac remodeling by* Miat*, we isolated RNAs from LV of WT and *Miat*-KO mice that had been continuously treated with Ang II or a saline control for 7 days and performed paired-end RNA sequencing. In the basal state, the KO mice displayed altered expressions of genes associated with a number of biological functions, including gene transcription, cell cycle, protein phosphorylation, DNA damage response, intracellular transduction, chromatin modification and RNA splicing (**Table S4,** *left*). With Ang II treatment, the number of genes with altered expression levels was much higher in each of these functional categories (**Table S4,** *right*). In addition, KEGG pathway analyses revealed that the Ang II-induced differentially expressed genes between WT and KO mice are highly enriched in the PI3K-Akt and MAPK signaling pathways (**Table S5**).

**Figure 5:**
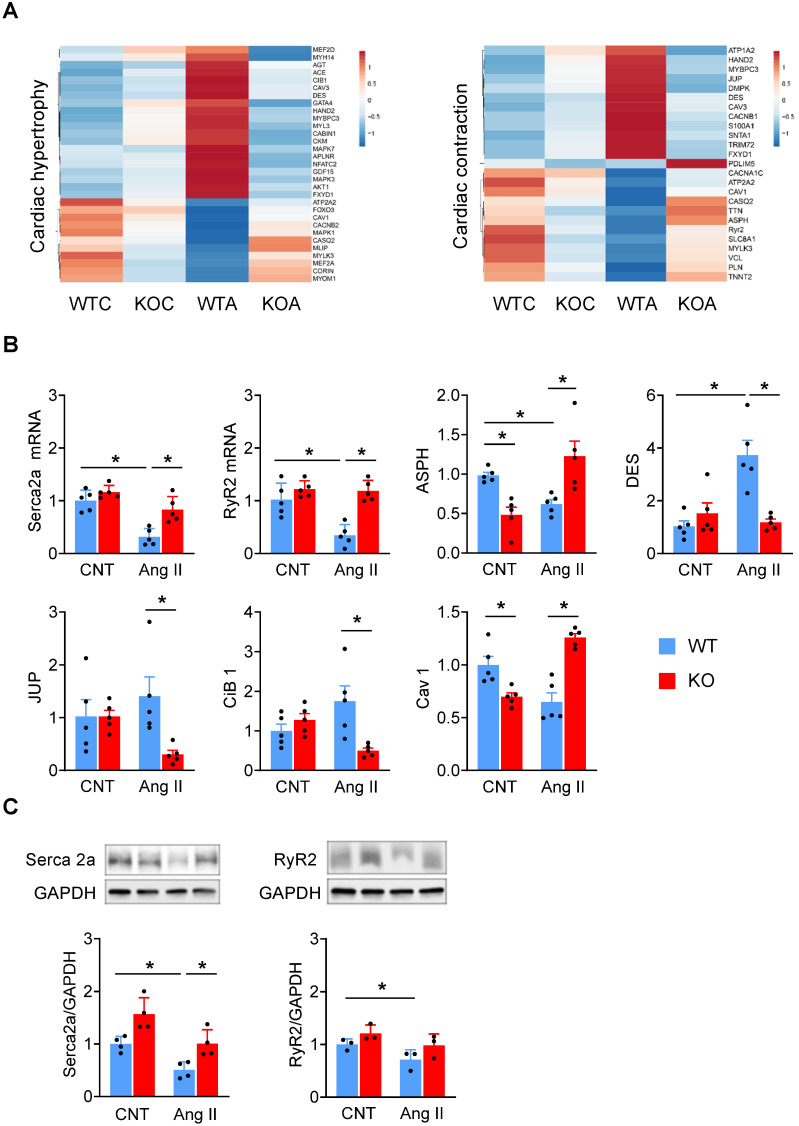
**Deletion of *Miat* blunts Ang II-induced hypertrophic gene program in the myocardium.** WT and *Miat*-KO mice were treated with Ang II (WTA, KOA) or saline control (WTC, KOC) for 7 days, then RNAs were isolated from LV and sequenced. (**A**) Heatmaps of genes associated with cardiac hypertrophy (*left* panel) and contractility (*right* panel). ATP2A2 = SERCA2a. (**B & C**) validation of gene expression levels by qRT-PCR (**B**) and Western blotting (**C**, n = 3-6). *p < 0.05, **p < 0.01.

Then we generated a heatmap of genes related to cardiac hypertrophy and contractility (**Figure 5A**). There were slight differences in the expression levels of these genes between WT and *Miat-*KO in the basal state. Ang II treatment led to drastic changes in their expression in the WT mice, including an elevation in the pro-hypertrophic genes (Mef2d, Gata4, Nfatc2, Hand2, Mapk7, Mapk3, Akt1, and Cib1) and a reduction in the anti-hypertrophic genes (Foxo3, Mlip, and Corin). However, these changes were largely diminished in the KO mice (**Figure 5A,** *left*). While Ang II treatment dramatically altered the expression of cardiac contractile genes in WT mice, including those involved in ion channels and calcium handling (Atp1a2, Cacnb1, S100a1, Fxyd1, Atp2a2, Casq2, Asph, RyR2, Slc8a1), myofibrils (Mybpc3, Pdlim5, Tnnt2), sarcolemma (Caveolin 1, Snta1, Mylk3) and cell junctions (Desmin, Jup), their changes are minimal in KO mice (**Figure 5A,** *right*). Furthermore, we confirmed the mRNA and protein expression of a number of key genes, including Serca2a (also known as Atp2a2), RyR2, Asph, Cib 1, Des, Jup and Cav 1 (**Figure 5B-C**). While the expression levels of Serca2a and RyR2 were comparable between WT and KO mice at baseline, Ang II treatment induced a marked downregulation of Serca2a and RyR2 in WT mice but not in KO mice (**Figure 5B-C, Figure S8**). Thus, our results clearly indicate that the Ang II-induced changes were blunted in *Miat*-KO mice, consistent with their attenuated hypertrophic response and enhanced contractility. Furthermore, in *Miat*-KD HL-1 cells, the level of Serca2a and RyR2 expression was better preserved than in HL-1 cells with Ang II treatment (**Figure S7**), and this protective effect was partially impaired by restoration of *Miat* expression (**Figure S7**).

In addition, bioinformatics analyses revealed that Ang II induced ~1000 significant splicing events in WT mice and strikingly only about 300 in *Miat*-KO mice, suggesting that the gene-splicing activities are significantly reduced in the absence of *Miat* (**Figure S9A**). We further analyzed the number of different types of splicing events. *Miat* KO led to a decreased number of splicing events in each type, most dramatically in skipped exon (SE) and alternative 3' splicing site (A3SS) (**Figure S9A**). Gene ontogeny (GO) analyses revealed that genes related to ventricular cardiac muscle tissue morphogenesis and cardiac muscle contraction were alternatively spliced due to the depletion of *Miat* (**Figure S9B**). In addition, the genes with changed constitution of isoforms regulate a number of biological processes, including heart contraction, mitochondria, cell junction and the oxidation-reduction process (**Table S6**). For example, phospholamban, a muscle-specific SR Ca^2+^-ATPase inhibitor highly expressed in cardiac muscle, was identified as two different isoforms in our dataset (**Figure S9C**). Although the function for each isoform is currently not known, our data demonstrate a significant decrease in the percentage of isoform 1 after Ang II treatment in WT mice (from 56% to 38%), but not in the KO mice (from 68% to 67%), suggesting that *Miat* is important for Ang II-induced alternative splicing of phospholamban. In addition, Cav 1.2 and Camk2b are key regulators of cardiac contraction and hypertrophy; while Ang II treatment resulted in decreased inclusion of Exon 10 in Cav 1.2 and increased inclusion of Exon 13 in Camk2b in the heart of WT mice, these Ang II-induced splicing alterations were blunted in KO mice (**Figure S9D-E**). Collectively, these data support that *Miat* regulates the expression and alternative splicing of genes related to hypertrophy and contractility.

### Deletion of *Miat* enhances the contractility of cardiomyocytes

**Figure 6:**
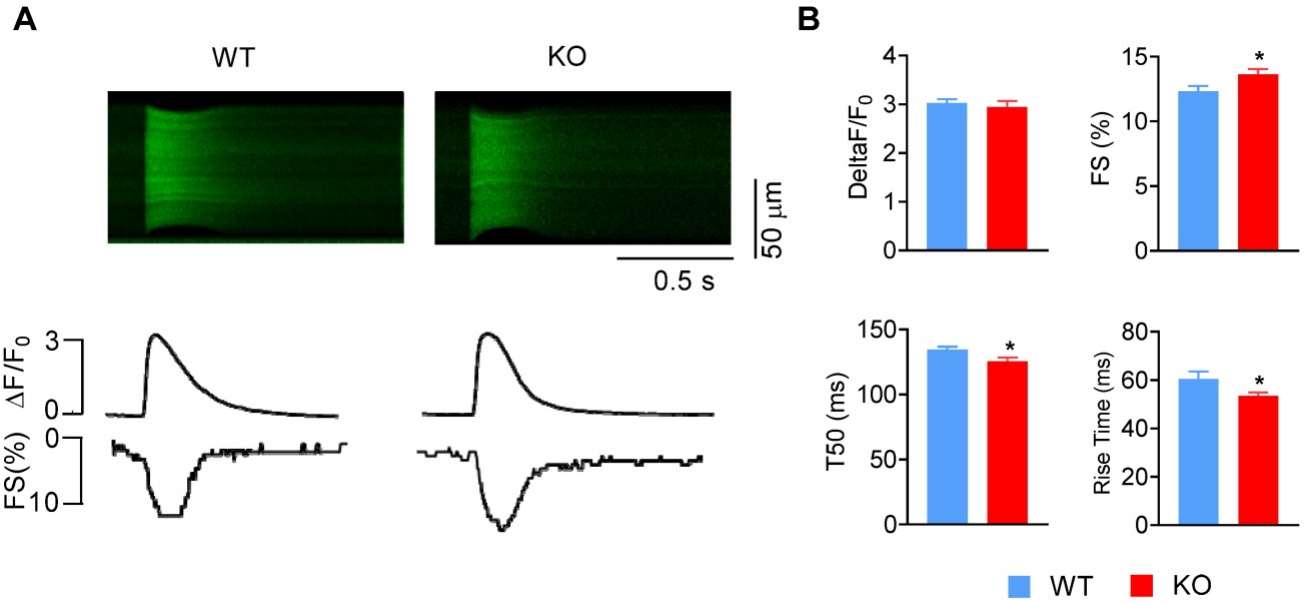
**Deletion of *Miat* increases cardiomyocyte contractility.** Cardiomyocytes were isolated from adult WT and *Miat*-KO mice. (**A**) Representative confocal line-scan images of Ca^2+^ transients (*upper* panels) along with their spatial averages and cell contractility by Fractional shortening (FS) (*lower* panels) in response to field stimulation (1 Hz). (**B**) Average calcium amplitude (ΔF/F_0_), cell fraction shortening, half-time of decay (T50) and the rise time of Ca^2+^ transient. *p < 0.05, **p < 0.01. n = 41-88.

Accordingly, we investigated whether *Miat* regulates the contractility of cardiomyocytes. Cardiomyocytes were isolated from adult WT and *Miat*-KO mice, and their fractional shortening (FS) and calcium transients were recorded (**Figure 6A-B**). KO cardiomyocytes display a slightly increased cell contractility, reduced rise time and half-time of decay (T50) of the Ca^2+^ transient, but comparable fold change of calcium amplitude, which suggests enhanced SR efficiency during Ca^2+^ cycling. The resting calcium concentration is unaltered (**Figure S10**). Collectively, these functional changes in KO cardiomyocytes are consistent with the altered gene expression and attenuated hypertrophic response in the KO mice.

## Discussion

In this study, we have identified a previously unrecognized function of *Miat* in adverse cardiac remodeling during mechanic stress. Genetic deletion of *Miat* attenuates pressure overload-induced pathological hypertrophy and heart failure, which is associated with a favorable global change in hypertrophic gene expression in the stressed myocardium and enhanced calcium handling and contractility of cardiomyocytes. Although several recent reports have suggested potential involvements of *Miat* in cardiac pathologies 23-25, 36, our study for the first time demonstrates that endogenous *Miat* suppresses cardiomyocyte contractile function to contribute to the development of pathological hypertrophy and heart failure.

We used two cardiac hypertrophy mouse models, revealing distinct pathophysiological conditions. The Ang II model features a decreased ventricular chamber size and impaired diastolic function, which mimics heart failure with preserved EF (HFpEF) in clinical settings. The TAC model indicates typical heart failure with reduced EF (HFrEF), characterized by dramatic decrease in EF and FS and a dilated ventricular chamber. Although ACE inhibitors 37 /ARBs 38 /ARNI 39, β-blockers 40 and aldosterone antagonists 41 have been proven to improve the clinical outcome of HFrEF, none of them have shown remarkably beneficial effects in the treatment of HFpEF. It is exciting that our data indicates ablation of *Miat* protects against both systolic and diastolic cardiac dysfunction. This has brought the clinical significance for *Miat* as a potential therapeutic target in HF, especially in HFpEF. Further studies are warranted to test the role of *Miat* in other HFpEF models.

Our results reveal that, while the expression levels of Serca2a and RyR2 are comparable between WT and *Miat*-KO mice in the basal state, Ang II treatment induces a marked downregulation of Serca2a and RyR2 in WT mice but not in KO mice, suggesting that endogenous *Miat* is responsible for Serca2a and RyR2 downregulation during the hypertrophic stress. The beneficial roles of Serca2a and RyR2 expression in cardiomyocyte calcium handling during cardiac hypertrophy and heart failure have been well established 42-44: while RyR2 activity results in rapid Ca^2+^-release from the sarcoplasmic reticulum (SR) and transient increase of intracellular Ca^2+^ concentration to trigger cell contraction, Serca2a removes Ca^2+^ from the sarcoplasm through Ca^2+^ reuptake into the SR to ensure cell relaxation. Knockout of Serca2a in mice leads to heart dysfunction 45, heterozygous Serca2a mice display accelerated heart failure under pressure overload 46, while cardiac-specific overexpression of a high Ca^2+^-affinity mutant of Serca2a attenuates pressure overload-induced cardiac hypertrophy 47. Similarly, knockout of RyR2 is embryonic lethal with abnormal cardiomyocytes 48, and mutation of Ryr2 results in early cardiac hypertrophy and lethality 49. Thus, our data support that *Miat* aggravates stress-induced Ca^2+^ mishandling through Serca2a and RyR2 downregulation. Notably, the better preserved Serca2a and RyR2 expression in *Miat*-KO mice precedes the alleviation of cardiac hypertrophy, suggesting a causal role of *Miat*-mediated Serca2a and RyR2 downregulation in the pathogenesis of cardiac hypertrophy.

We found attenuated cardiomyocyte apoptosis and interstitial fibrosis in the heart of KO mice, which is consistent with the enhanced systolic and diastolic function in these mice and suggests that depletion of *Miat* suppresses the cardiac remodeling during cardiac hypertrophy and failure. In addition, we observed a better-preserved capillary density in *Miat*-KO mice. While it is well known that cardiac hypertrophic pathology is associated with reduced vascular density, dysfunctional coronary vasculature has also been shown to contribute to the pathogenesis of cardiac hypertrophy 50. Interestingly, Yan et al. showed that knockdown of *Miat* attenuates diabetes-associated deregulation of retinal microvasculature by protecting high glucose-induced apoptosis of endothelial cells 26. Although the biological context in our study is different, and our RNA-seq data from the whole heart did not specifically indicate a prominent molecular signature of neovascularization, potential effects of *Miat* in the coronary circulation and function cannot be ruled out and warrants further investigation.

In our RNA-seq dataset from Ang II-stressed hearts, MMP2 and galectin-3 levels were lowered, while myeloperoxidase, TNF-α, and IL-6 levels were unaltered in *Miat* knockout mice compared to WT mice. Although galectin-3 is predominantly expressed by activated macrophages and a promising biomarker for patients with heart failure, it does not appear to affect the survival, systolic and diastolic dysfunction, cardiac fibrosis, and cardiomyocyte hypertrophy in the pressure-overloaded heart 51. On the other hand, targeted deletion of MMP2 ameliorates myocardial remodeling in mice with chronic pressure overload 52, thus the reduced MMP2 expression in *Miat*-KO mice may have also potentially contributed to the attenuated cardiac fibrosis and remodeling.

Notably, we found that Ang II induces a marked increase in the splicing events in the myocardium of WT mice, which is largely blunted in *Miat*-KO mice. Although *Miat* has been shown to be involved in RNA splicing in neuronal and renal cells 18, 53-55, such as by interaction with the Qki and Srsf1 splicing factors to alter the splicing of Disc1 and ErbB4 in schizophrenia 56, 57, our study for the first time shows that *Miat* profoundly contributes to the RNA splicing in hypertrophic hearts. Recently, the roles of Qki and Srsf1 in cardiac 58, 59 and vascular 60-62 biology are increasingly appreciated. Further studies are underway to investigate whether *Miat* regulates cardiac RNA splicing through Qki and Srsf1 and what the functions of our identified splicing isoforms are in the pathogenesis of cardiac hypertrophy.

In conclusion, our results suggest that in the stressed heart, endogenous *Miat* jeopardizes cardiomyocyte calcium handling and contractile function, contributing to adverse myocardial remodeling and hypertrophy. Thus, in-depth investigation of *Miat* may lead to identification of new therapeutic targets.

## Supplementary Material

Supplementary figures and tables.
