# Supplementary material for: Ablation of lncRNA Miat attenuates pathological hypertrophy and heart failure
Source: Theranostics. 2021 Jul 6;11(16):7995–8007. doi: 10.7150/thno.50990 (PMC8315059; doi:10.7150/thno.50990)
Supplement: Supplementary file 1 — Supplementary figures and tables. [file thnov11p7995s1.pdf]

Ablation of lncRNA *Miat* attenuates pathological hypertrophy and heart failure

Liu Yang[1,2,3], Jianxin Deng[1], Wenxia Ma[1], Aijun Qiao[1], Shiyue Xu[1], Yang Yu[2], Chan Boriboun[1], Xiang Kang[1], Dunzheng Han[1], Patrick Ernst[1], Lufang Zhou[1,4], Jiawei Shi[1], Eric Zhang[1], Tao-Sheng Li[5], Hongyu Qiu[6], Shinichi Nakagawa[7], Seth Blackshaw[8], Jianyi Zhang[1], Gangjian Qin[1,2,#]

Supplementary Information

Supplementary Figures

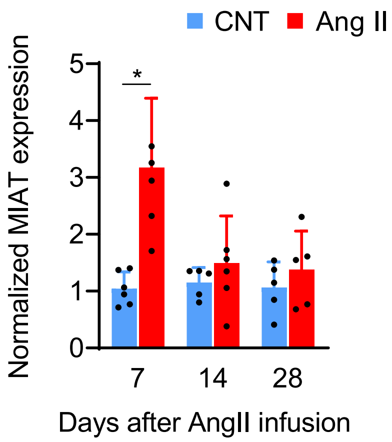

Figure S1. *Miat* expression is increased in the myocardium after Ang II infusion for 7 days. *Miat* expression was analyzed by qRT-PCR in the LV tissue of WT mice after saline (CNT) or Ang II (2 mg/kg BW/day) infusion via mini-osmotic pump for 7, 14 or 28 days. The expression levels were normalized to 18S, and expressed relative to the levels in the mice with saline treatment for 7 days. n = 5 per group. *p < 0.05 vs. CNT.

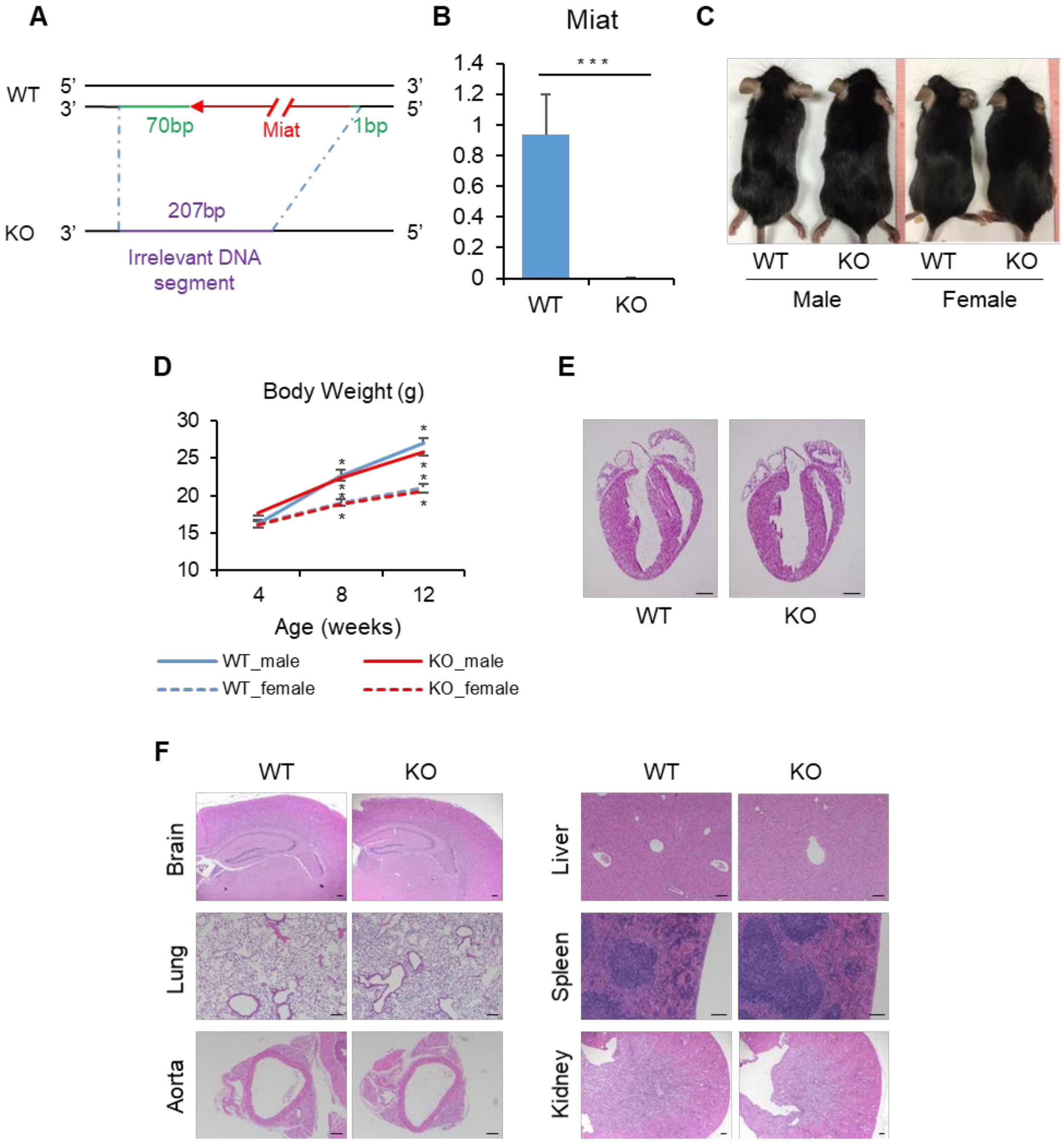

Figure S2. *Miat*-KO mice do not exhibit an obvious developmental defect. (**A**) Schema of genetic deletion of *Miat*. (**B**) qRT-PCR analysis of *Miat* in the heart of WT and *Miat*-KO mice, n = 4 per group. ***p < 0.001. (**C-D**) Gross examination (**C**) and body weight (**D**) of WT and *Miat*-KO mice, n = 10 per group. **p < 0.01, ***p < 0.001 vs. the level at Week 4. (**E-F**) Representative images for HE staining of hearts (**E**, scale bar = 1 mm) and other major organs (**F,** scale bar = 100 μm) in WT and *Miat*-KO mice.

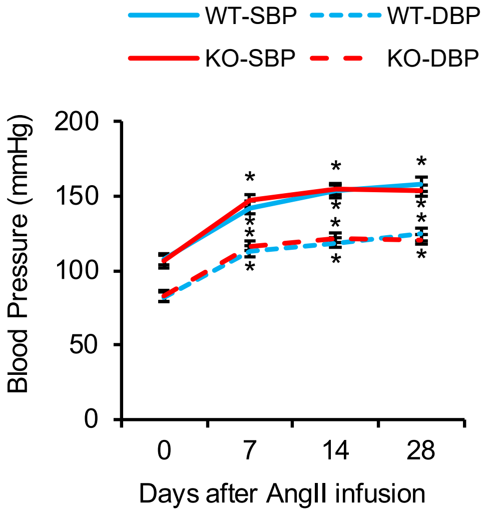

Figure S3. Ang II-induced hypertension is similar in WT and *Miat*-KO mice. Blood pressure of WT and KO mice at the indicated time points of Ang II treatment. SBP, systolic blood pressure. DBP, diastolic blood pressure. n = 11 per group. *p < 0.05 vs. Day 0.

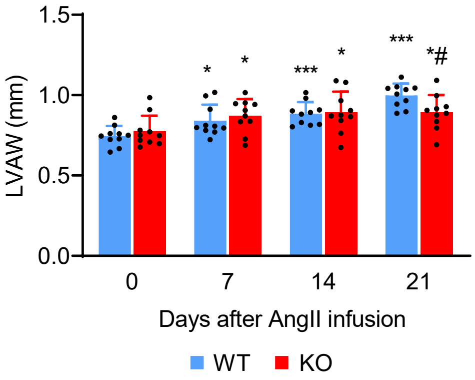

Figure S4. Ang II-induced LV wall thickening is attenuated in *Miat*-KO mice. *Miat*-KO mice and WT littermates were treated with Ang II (2 mg/Kg BW/day) via a subcutaneously-implanted mini-osmotic pump. The thicknesses of LV anterior wall (LVAW) were measured via echocardiography at the indicated time points after initiation of Ang II infusion. *p < 0.05, ***p < 0.001 vs. Day 0. #p < 0.05 vs. WT.

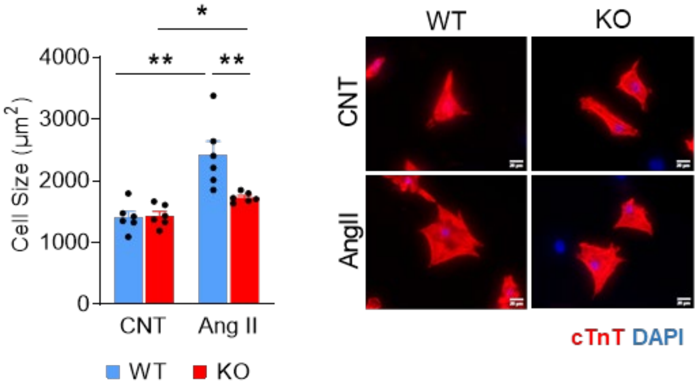

Figure S5. Deletion of *Miat* attenuates Ang II-induced hypertrophy of cardiomyocytes. Cardiomyocytes were isolated from neonatal WT or *Miat*-KO mice and treated with Ang II (200 nM) or PBS (CNT) for 48 h, then stained with anti-cardiac troponin T (cTnT, red) and DAPI (blue). The area per cell was analyzed with NIH Image J and averaged. *p < 0.05, **p < 0.01. Scale bar = 20 µm.

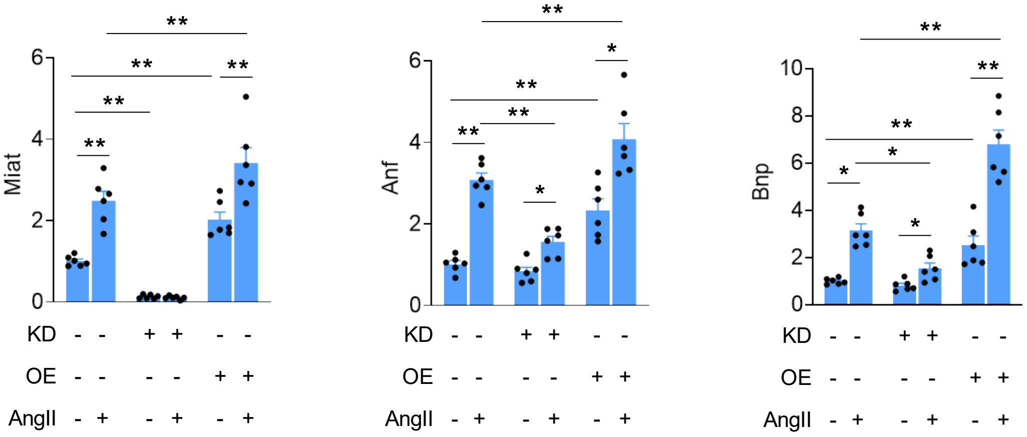

Figure S6. Knockdown (KD) of *Miat* attenuates, and overexpression (OE) of *Miat* aggravates, Ang II-induced hypertrophic gene program in HL-1 cells. HL-1 cells were infected with lentiviral vector coding for *Miat* shRNA (KD +) or control shRNA (KD –) and 72 h later, transfected with a plasmid coding for mouse *Miat* (OE +) or an empty plasmid (OE –). After overnight, the cells were treated with Ang II (200 nM, +) or PBS (–) for 24 h. The mRNA levels of *Miat*, Anf and Bnp were evaluated via qRT-PCR, normalized to 18S, and expressed relative to the values of KD (–) group. *p < 0.05, **p < 0.01.

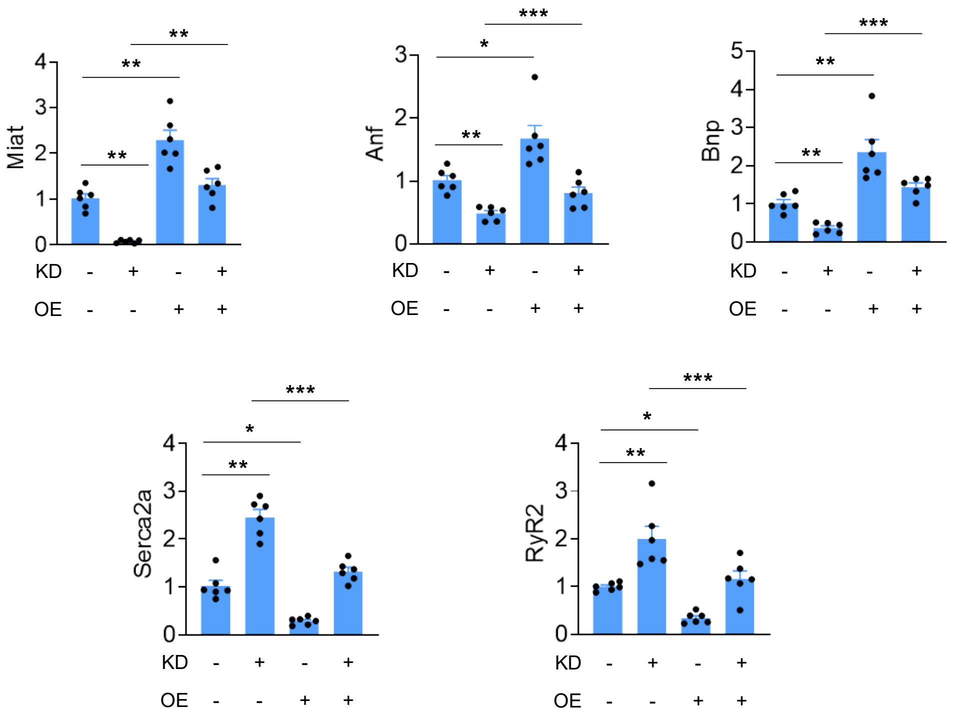

Figure S7. The attenuation of Ang II-induced hypertrophic gene program in *Miat*-KD HL-1 cells is reversed by *Miat* overexpression. HL-1 cells were infected with lentiviral vector coding for *Miat* shRNA (KD +) or control shRNA (KD –) and 72 h later, transfected with a plasmid coding for mouse *Miat* (OE +) or an empty plasmid (OE –). After overnight, the cells were treated with Ang II (200 nM) for 24 h. The mRNA levels of *Miat*, Anf, Bnp, Serca2a and RyR2 were evaluated via qRT-PCR, normalized to 18S, and expressed relative to the values of KD (–) group. *p < 0.05, **p < 0.01, ***p < 0.001.

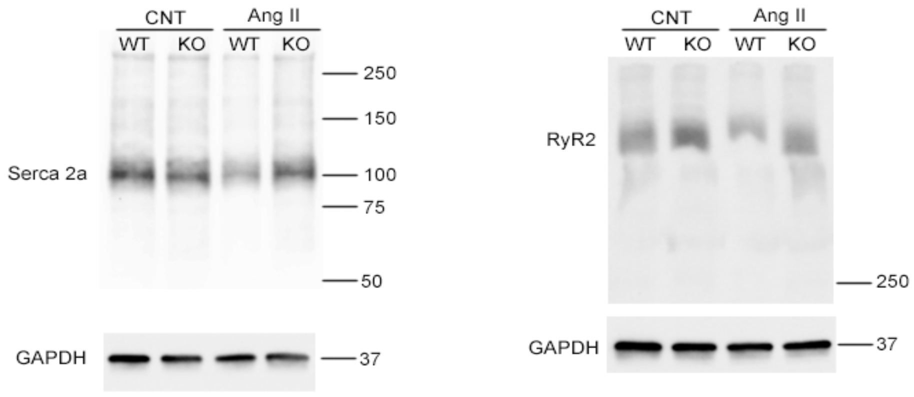

Figure S8. The original Western blots in Fig 5C.

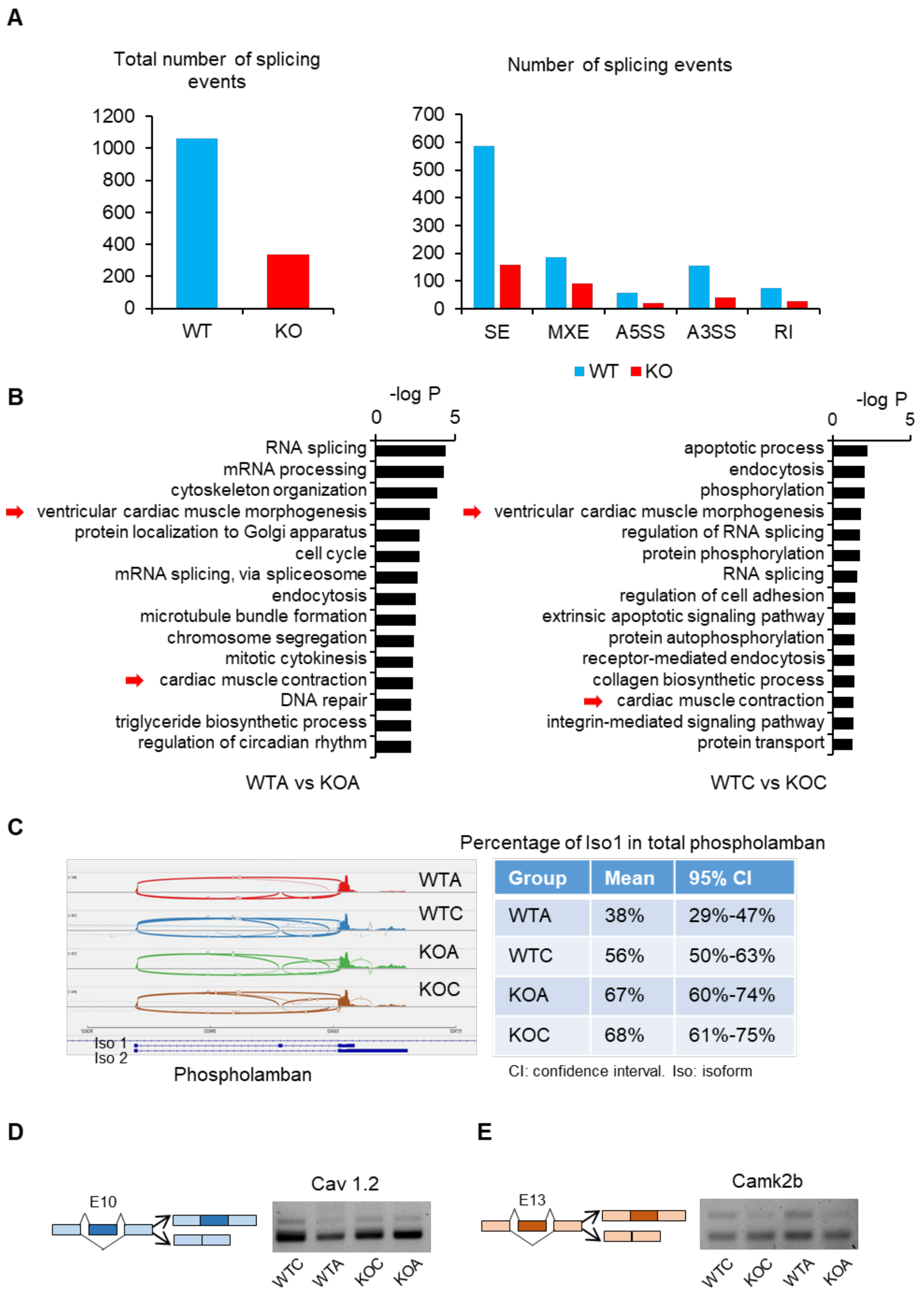

Figure S9. Ang II-induced alternative splicing of cardiac genes is markedly attenuated in *Miat*-KO mice. Both WT and KO mice were treated with Ang II (WTA, KOA) or saline (WTC,

KOC) via mini-pump for 7 days. Then RNAs were isolated from left ventricles and sequenced. (**A**) Total number of splicing events. SE, skipped exon; MXE, mutually exclusive exons; A5SS, alternative 5' splice site; A3SS, alternative 3' splice site; RI, retained intron. (**B**) Top 15 biological processes in GO analyses of genes with significant alternative splicing events. (**C**) Phospholamban isoform constitution. (**D-E**) Semiquantitative RT-PCR analysis showing splicing alterations of Cav 1.2 (**D**) and Camk2b (**E**).

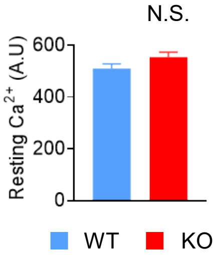

Figure S10. Deletion of *Miat* does not change resting calcium concentration in cardiomyocytes. Cardiomyocytes were isolated from adult WT and *Miat*-KO mice, and resting intracellular Ca^{2+} levels (F0), n = 140 for WT, n = 114 for KO. N.S., not significant vs. WT.

Supplementary Tables

Table S1. List of primers

	Forward primer	Reverse primer
**For assessment of gene expression with qRT-PCR**		
Miat	5’-GGGAGGTGTATAAAGTGAGAAGCT-3’	5’-GTATCCCAAGGAATGAAGTCTGTCT-
Bnp	5’-TGGGAGGTCACTCCTATCCT-3’	5’-GGCCATTTCCTCCGACTT-3’
Anf	5’-TCTTCCTCTTGGCCTTT-3’	5’-CCAGGTGGTCTAGCAGGTTC-3’
18S	5’- CACGGCCGGTACAGTGAAA-3’	5’- AGAGGAGCGAGCGACCAA-3’
β-actin	5’-ATGTGGATCAGCAAGCAGGA-3’	5’-AAGGGTGTAAAACGCAGCTCA-3’
**For validation of splicing alterations with semiquantitative RT-PCR**		
Cav 1.2	5’-CCTCATCGTCATTGGGAGCAT-3’	5’- TGATGAAGGTCCACAGCAGG-3’
Camk2b	5’-AGCCATCCTCACCACTATGC-3’	5’-ACTCCATCTGCTTTCTTGTTGAG-3’

Table S2. Echocardiographic analyses in Ang II infusion model

	WT		KO	
Treatment	CNT	AngII	CNT	AngII
LVAW (mm)	0.80 ± 0.06	1.05 ± 0.10**	0.80 ± 0.02	0.93 ± 0.13*#
LVPW (mm)	0.79 ± 0.08	1.13 ± 0.13**	0.81 ± 0.04	0.92 ± 0.19*#
LVID; d (mm)	3.74 ± 0.29	3.21 ± 0.27*	3.68 ± 0.32	3.63 ± 0.54#
LVID; s (mm)	2.31 ± 0.27	1.76 ± 0.23*	2.31 ± 0.20	2.18 ± 0.55#
LV Vol; d (μL)	51.37 ± 5.45	39.46 ± 4.89*	49.66 ± 4.15	50.98 ± 8.51#
LV Vol; s (μL)	17.72 ± 4.77	11.43 ± 3.08*	16.28 ± 3.33	17.12 ± 4.72#
EF (%)	67.94 ± 3.48	74.42 ± 3.19*	67.70 ± 1.76	69.70 ± 2.98#
FS (%)	37.04 ± 2.73	43.39 ± 6.07*	36.96 ± 1.39	38.67 ± 3.12#
E/E'	34.12 ± 2.42	46.18 ± 5.25*	35.74 ± 1.59	40.05 ± 4.48*#
IVRT (ms)	15.88 ± 2.96	21.86 ± 2.87*	16.80 ± 1.47	19.59 ± 1.79*#
SV (μL)	38.51 ± 2.98	30.61 ± 3.85*	37.28 ± 6.23	36.67 ± 6.87#
CO (mL/min)	22.39 ± 3.12	16.62 ± 2.38*	22.21 ± 1.56	21.14 ± 3.98#

*p < 0.05, **p < 0.01 vs. CNT. #p < 0.05 vs. WT.

Table S3. Echocardiographic analyses in TAC model

	WT		KO	
Treatment	Sham	TAC	Sham	TAC
LVAW (mm)	0.78 ± 0.04	0.98 ± 0.07**	0.76 ± 0.06	0.85 ± 0.03*##
LVPW (mm)	0.83 ± 0.07	1.03 ± 0.12**	0.79 ± 0.06	0.82 ± 0.03##
LVID; d (mm)	3.45 ± 0.32	3.95 ± 0.44*	3.41 ± 0.31	3.30 ± 0.14##
LVID; s (mm)	2.26 ± 0.32	3.04 ± 0.45**	2.19 ± 0.42	2.19 ± 0.35#
LV Vol; d (μL)	47.35 ± 6.82	56.48 ± 2.36**	46.78 ± 4.56	44.27 ± 4.64##
LV Vol; s (μL)	17.71 ± 5.95	24.08 ± 3.51**	16.29 ± 3.52	14.85 ± 3.67##
EF (%)	68.72 ± 4.41	49.74 ± 7.77**	69.25 ± 4.32	69.45 ± 3.91##
FS (%)	39.09 ± 2.08	25.63 ± 4.60*	38.14 ± 3.47	38.22 ± 2.95#

*p < 0.05, **p < 0.01 vs. Sham. #p < 0.05, ##p < 0.01 vs. WT.

Table S4. Top 15 biological processes in the GO analyses

KOC vs. WTC			KOA vs. WTA		
Name	Count	p value	Name	Count	p value
regulation of transcription	283	1.95E-08	regulation of transcription	720	1.54E-04
cell cycle	84	1.80E-04	cell cycle	275	7.57E-19
protein phosphorylation	73	3.85E-03	protein transport	262	3.26E-17
cellular response to DNA damage stimulus	68	4.41E-06	protein phosphorylation	244	3.07E-13
intracellular signal transduction	55	2.34E-03	apoptotic process	214	1.25E-06
covalent chromatin modification	54	4.15E-08	cell adhesion	186	1.34E-06
regulation of gene expression	53	5.75E-03	cell division	178	2.65E-15
cell division	51	4.04E-03	cellular response to DNA damage stimulus	177	1.09E-09
mRNA processing	48	8.78E-04	metabolic process	157	5.92E-03
protein ubiquitination	47	1.38E-02	intracellular signal transduction	155	5.54E-06
regulation of cell proliferation	46	5.00E-02	lipid metabolic process	153	1.27E-02
regulation of apoptotic process	43	2.20E-02	protein ubiquitination	151	4.35E-08
mitotic nuclear division	41	2.47E-03	DNA repair	140	2.44E-09
RNA splicing	39	6.14E-04	translation	134	1.77E-02
small GTPase mediated signal transduction	38	8.02E-04	positive regulation of gene expression	131	3.28E-02

Gene ontology (GO) analyses showing top 15 biological processes with differences of gene expression levels > 2 folds between WT and *Miat*-KO mice after Saline (WTC, KOC) or Ang II (WTA, KOA) treatment for 7 days.

Table S5. Top 10 KEGG pathways in the GO analyses

KOC vs. WTC			KOA vs. WTA		
Name	Count	p value	Name	Count	p value
MAPK signaling pathway	36	2.80E-03	Metabolic pathways	387	2.94E-02
Ras signaling pathway	30	2.19E-02	PI3K-Akt signaling pathway	123	3.47E-03
Focal adhesion	28	1.87E-02	Endocytosis	107	7.39E-06
Regulation of actin cytoskeleton	28	2.61E-02	MAPK signaling pathway	97	2.88E-04
Rap1 signaling pathway	28	2.75E-02	Focal adhesion	94	1.41E-07
Chemokine signaling pathway	25	4.88E-02	Regulation of actin cytoskeleton	86	1.22E-04
Cell cycle	23	8.01E-04	Rap1 signaling pathway	86	1.47E-04
Wnt signaling pathway	23	4.33E-03	Ras signaling pathway	83	6.49E-03
Thyroid hormone signaling pathway	21	1.57E-03	Insulin signaling pathway	77	5.27E-11
Ubiquitin mediated proteolysis	21	1.89E-02	Protein processing in endoplasmic reticulum	73	2.69E-05

Gene ontology (GO) analyses showing top 10 KEGG pathways with differences of gene expression levels > 2 folds between WT and *Miat*-KO mice after Saline (WTC, KOC) or Ang II (WTA, KOA) treatment for 7 days.

Table S6 List of genes with altered constitution of isoforms

Category	Ensemble Gene ID	Gene Symbol	Full Name	Ensemble Isoform ID	WTAvsWTB	KOAvsWTA	KOAvsKOB	KOBvsWTB
Regulation of the force of heart	ENSMUSG00000029467	Atp2a2	ATPase, Ca++ transporting, cardiac muscle, slow twitch 2	ENSMUST00000177974	-0.28	0.26	0.01	-0.03
	ENSMUSG00000038583	Pln	phospholamban	ENSMUST00000163319	-0.18	0.29	-0.01	0.12
	ENSMUSG00000007097	Atp1a2	ATPase, Na+/K+ transporting, alpha 2 polypeptide	ENSMUST00000085913	-0.5	0.41	0.13	-0.22
	ENSMUSG00000007097	Atp1a2	ATPase, Na+/K+ transporting, alpha 2 polypeptide	ENSMUST00000097464	0.4	-0.32	-0.07	0.15
contraction (calcium channel,	ENSMUSG00000028207	Asph	aspartate-beta-hydroxylase	ENSMUST00000103004	0.27	-0.36	-0.12	0.03
	ENSMUSG00000002279	Lmf1	lipase maturation factor 1	ENSMUST00000137201	0.5	-0.42	-0.02	0.1
	ENSMUSG00000029103	Lrpap1	low density lipoprotein receptor-related protein associated protein 1	ENSMUST00000030986	-0.41	0.36	0.27	-0.32
	ENSMUSG00000022037	Clu	clusterin	ENSMUST00000022616	0.36	-0.06	0.28	0.03
sarcoplasmic reticulum related)	ENSMUSG00000004846	Plod3	procollagen-lysine, 2-oxoglutarate 5-dioxygenase 3	ENSMUST00000144784	0.39	-0.36	-0.04	0.06
	ENSMUSG00000053768	Chchd3	coiled-coil-helix-coiled-coil-helix domain containing 3	ENSMUST00000143206	0.45	-0.33	0.07	0.05
	ENSMUSG00000053768	Chchd3	coiled-coil-helix-coiled-coil-helix domain containing 3	ENSMUST00000125264	-0.24	0.22	-0.08	0.06
	ENSMUSG00000038967	Pdk2	pyruvate dehydrogenase kinase, isoenzyme 2	ENSMUST00000038431	0.32	-0.06	-0.01	0.27
	ENSMUSG00000038967	Pdk2	pyruvate dehydrogenase kinase, isoenzyme 2	ENSMUST00000141712	-0.35	0.03	-0.03	-0.29
	ENSMUSG00000029776	Hibadh	3-hydroxyisobutyrate dehydrogenase	ENSMUST00000031788	-0.62	0.64	-0.01	0.03
	ENSMUSG00000029776	Hibadh	3-hydroxyisobutyrate dehydrogenase	ENSMUST00000155948	0.61	-0.66	-0.01	-0.04
	ENSMUSG00000020163	Uqcr11	ubiquinol-cytochrome c reductase, complex III subunit XI	ENSMUST00000020372	-0.36	0.18	-0.08	-0.1
	ENSMUSG00000020163	Uqcr11	ubiquinol-cytochrome c reductase, complex III subunit XI	ENSMUST00000141683	0.39	-0.19	0.07	0.14
	ENSMUSG00000020849	Ywhae	tyrosine 3-monooxygenase/tryptophan 5-monooxygenase activation protein, epsilon polypeptide	ENSMUST00000067664	-0.56	0.59	0.06	-0.03
	ENSMUSG00000020849	Ywhae	tyrosine 3-monooxygenase/tryptophan 5-monooxygenase activation protein, epsilon polypeptide	ENSMUST00000134745	0.54	-0.53	0	0.01
	ENSMUSG00000025825	Iscu	iron-sulfur cluster assembly enzyme	ENSMUST00000026937	-0.39	0.35	0.11	-0.15
	ENSMUSG00000022037	Clu	clusterin	ENSMUST00000022616	0.36	-0.06	0.28	0.03
	ENSMUSG00000014606	Slc25a11	solute carrier family 25 (mitochondrial carrier oxoglutarate carrier), member 11	ENSMUST00000139638	-0.21	0.04	-0.05	-0.13
Mitochondrion	ENSMUSG00000014606	Slc25a11	solute carrier family 25 (mitochondrial carrier oxoglutarate carrier), member 11	ENSMUST00000136383	0.34	-0.26	-0.14	0.22
	ENSMUSG00000024359	Hspa9	heat shock protein 9	ENSMUST00000025217	-0.58	0.37	-0.07	-0.14
	ENSMUSG00000040048	Ndufb10	NADH dehydrogenase (ubiquinone) 1 beta subcomplex, 10	ENSMUST00000129451	0.39	-0.21	0.16	0.02
	ENSMUSG00000106918	Mrpl33	mitochondrial ribosomal protein L33	ENSMUST00000031024	0.54	-0.23	0.1	0.21
	ENSMUSG00000032563	Mrpl3	mitochondrial ribosomal protein L3	ENSMUST00000142424	0.53	-0.13	-0.11	0.51
	ENSMUSG00000031969	Acad8	acyl-Coenzyme A dehydrogenase family, member 8	ENSMUST00000151075	0.67	-0.66	-0.12	0.13
	ENSMUSG00000038583	Pln	phospholamban	ENSMUST00000163319	-0.18	0.29	-0.01	0.12
	ENSMUSG00000027774	Gfm1	G elongation factor, mitochondrial 1	ENSMUST00000077271	-0.61	0.49	0.19	-0.31
	ENSMUSG00000027774	Gfm1	G elongation factor, mitochondrial 1	ENSMUST00000161009	0.59	-0.6	-0.16	0.15
	ENSMUSG00000028959	Fastk	Fas-activated serine/threonine kinase	ENSMUST00000123144	0.33	-0.36	-0.07	0.04
	ENSMUSG00000046598	Bdh1	3-hydroxybutyrate dehydrogenase, type 1	ENSMUST00000149039	0.43	-0.39	0	0.03
	ENSMUSG00000026701	Prdx6	peroxiredoxin 6	ENSMUST00000192639	-0.32	0.14	0.02	-0.19
	ENSMUSG00000026701	Prdx6	peroxiredoxin 6	ENSMUST00000051925	0.47	-0.37	-0.13	0.23
	ENSMUSG00000053898	Ech1	enoyl coenzyme A hydratase 1, peroxisomal	ENSMUST00000066264	0.33	-0.33	0.08	-0.08
	ENSMUSG00000053898	Ech1	enoyl coenzyme A hydratase 1, peroxisomal	ENSMUST00000143708	-0.21	0.04	0	-0.17
	ENSMUSG00000027406	Idh3b	isocitrate dehydrogenase 3 (NAD+) beta	ENSMUST00000149843	0.24	-0.22	0.01	0
	ENSMUSG00000025006	Sorbs1	sorbin and SH3 domain containing 1	ENSMUST00000099467	0.6	-0.54	0.02	0.04
	ENSMUSG00000030870	Ubfd1	ubiquitin family domain containing 1	ENSMUST00000033158	-0.37	0.31	0.1	-0.15
	ENSMUSG00000030870	Ubfd1	ubiquitin family domain containing 1	ENSMUST00000124733	0.57	-0.55	-0.03	0.04
	ENSMUSG00000028273	Pdlim5	PDZ and LIM domain 5	ENSMUST00000029941	0.26	-0.27	-0.02	0.01
	ENSMUSG00000071644	Eef1g	eukaryotic translation elongation factor 1 gamma	ENSMUST00000128626	0.32	-0.11	0.03	0.18
Cell junction	ENSMUSG00000015656	Hspa8	heat shock protein 8	ENSMUST00000153847	-0.39	0.28	0.29	-0.4
	ENSMUSG00000015656	Hspa8	heat shock protein 8	ENSMUST00000117557	0.31	-0.24	0.07	-0.01
	ENSMUSG00000020849	Ywhae	tyrosine 3-monooxygenase/tryptophan 5-monooxygenase activation protein, epsilon polypeptide	ENSMUST00000067664	-0.56	0.59	0.06	-0.03
	ENSMUSG00000020849	Ywhae	tyrosine 3-monooxygenase/tryptophan 5-monooxygenase activation protein, epsilon polypeptide	ENSMUST00000134745	0.54	-0.53	0	0.01
	ENSMUSG00000026701	Prdx6	peroxiredoxin 6	ENSMUST00000192639	-0.32	0.14	0.02	-0.19
	ENSMUSG00000026701	Prdx6	peroxiredoxin 6	ENSMUST00000051925	0.47	-0.37	-0.13	0.23
	ENSMUSG00000029776	Hibadh	3-hydroxyisobutyrate dehydrogenase	ENSMUST00000031788	-0.62	0.64	-0.01	0.03
	ENSMUSG00000029776	Hibadh	3-hydroxyisobutyrate dehydrogenase	ENSMUST00000155948	0.61	-0.66	-0.01	-0.04
	ENSMUSG00000020163	Uqcr11	ubiquinol-cytochrome c reductase, complex III subunit XI	ENSMUST00000020372	-0.36	0.18	-0.08	-0.1
	ENSMUSG00000020163	Uqcr11	ubiquinol-cytochrome c reductase, complex III subunit XI	ENSMUST00000141683	0.39	-0.19	0.07	0.14
	ENSMUSG00000004846	Plod3	procollagen-lysine, 2-oxoglutarate 5-dioxygenase 3	ENSMUST00000144784	0.39	-0.36	-0.04	0.06
	ENSMUSG00000028207	Asph	aspartate-beta-hydroxylase	ENSMUST00000103004	0.27	-0.36	-0.12	0.03
	ENSMUSG00000040048	Ndufb10	NADH dehydrogenase (ubiquinone) 1 beta subcomplex, 10	ENSMUST00000129451	0.39	-0.21	0.16	0.02
Oxidation-reduction process	ENSMUSG00000026701	Prdx6	peroxiredoxin 6	ENSMUST00000192639	-0.32	0.14	0.02	-0.19
	ENSMUSG00000026701	Prdx6	peroxiredoxin 6	ENSMUST00000051925	0.47	-0.37	-0.13	0.23
	ENSMUSG00000046598	Bdh1	3-hydroxybutyrate dehydrogenase, type 1	ENSMUST00000149039	0.43	-0.39	0	0.03

	ENSMUSG00000031969	Acad8	acyl-Coenzyme A dehydrogenase family, member 8	ENSMUST00000151075	0.67	-0.66	-0.12	0.13
	ENSMUSG00000020849	Ywhae	tyrosine 3-monooxygenase/tryptophan 5-monooxygenase activation protein, epsilon polypeptide	ENSMUST00000067664	-0.56	0.59	0.06	-0.03
	ENSMUSG00000020849	Ywhae	tyrosine 3-monooxygenase/tryptophan 5-monooxygenase activation protein, epsilon polypeptide	ENSMUST00000134745	0.54	-0.53	0	0.01
	ENSMUSG00000027406	Idh3b	isocitrate dehydrogenase 3 (NAD+) beta	ENSMUST00000149843	0.24	-0.22	0.01	0
	ENSMUSG00000060675	Pla2g16	phospholipase A2, group XVI	ENSMUST00000025925	-0.54	0.27	0.17	-0.43
	ENSMUSG00000060675	Pla2g16	phospholipase A2, group XVI	ENSMUST00000141887	0.79	-0.74	-0.21	0.26
GTPase activity	ENSMUSG00000029713	Gnb2	guanine nucleotide binding protein (G protein), beta 2	ENSMUST00000167225	0.46	-0.32	0.07	0.07
	ENSMUSG00000031748	Gnao1	guanine nucleotide binding protein, alpha O	ENSMUST00000144451	0.36	-0.34	0	0.01
	ENSMUSG00000026202	Tuba4a	tubulin, alpha 4A	ENSMUST00000187103	-0.28	0.26	-0.06	0.04
	ENSMUSG00000027774	Gfm1	G elongation factor, mitochondrial 1	ENSMUST00000077271	-0.61	0.49	0.19	-0.31
	ENSMUSG00000027774	Gfm1	G elongation factor, mitochondrial 1	ENSMUST00000161009	0.59	-0.6	-0.16	0.15
Cytoskeleton	ENSMUSG00000020483	Dynll2	dynein light chain LC8-type 2	ENSMUST00000178105	0.57	-0.46	0.16	-0.05
	ENSMUSG00000020483	Dynll2	dynein light chain LC8-type 2	ENSMUST00000020775	-0.58	0.46	-0.22	0.1
	ENSMUSG00000031813	Mvb12a	multivesicular body subunit 12A	ENSMUST00000212326	0.46	-0.48	-0.01	-0.01
	ENSMUSG00000023150	Ivns1abp	influenza virus NS1A binding protein	ENSMUST00000023918	-0.51	0.4	0.21	-0.31
	ENSMUSG00000023150	Ivns1abp	influenza virus NS1A binding protein	ENSMUST00000111887	0.44	-0.5	-0.1	0.04
	ENSMUSG00000027238	Frmd5	FERM domain containing 5	ENSMUST00000121219	0.42	-0.39	0.02	0.01
	ENSMUSG00000030782	Tgfb1i1	transforming growth factor beta 1 induced transcript 1	ENSMUST00000166755	0.29	-0.25	0.02	0.03
	ENSMUSG00000025006	Sorbs1	sorbin and SH3 domain containing 1	ENSMUST00000099467	0.6	-0.54	0.02	0.04
	ENSMUSG00000026202	Tuba4a	tubulin, alpha 4A	ENSMUST00000187103	-0.28	0.26	-0.06	0.04
Metal-binding	ENSMUSG00000029467	Atp2a2	ATPase, Ca++ transporting, cardiac muscle, slow twitch 2	ENSMUST00000177974	-0.28	0.26	0.01	-0.03
	ENSMUSG00000028273	Pdlim5	PDZ and LIM domain 5	ENSMUST00000029941	0.26	-0.27	-0.02	0.01
	ENSMUSG00000031748	Gnao1	guanine nucleotide binding protein, alpha O	ENSMUST00000144451	0.36	-0.34	0	0.01
	ENSMUSG00000025825	Iscu	iron-sulfur cluster assembly enzyme	ENSMUST00000026937	-0.39	0.35	0.11	-0.15
	ENSMUSG00000004846	Plod3	procollagen-lysine, 2-oxoglutarate 5-dioxygenase 3	ENSMUST00000144784	0.39	-0.36	-0.04	0.06
	ENSMUSG00000028207	Asph	aspartate-beta-hydroxylase	ENSMUST00000103004	0.27	-0.36	-0.12	0.03
	ENSMUSG00000027406	Idh3b	isocitrate dehydrogenase 3 (NAD+) beta	ENSMUST00000149843	0.24	-0.22	0.01	0
	ENSMUSG00000007097	Atp1a2	ATPase, Na+/K+ transporting, alpha 2 polypeptide	ENSMUST00000085913	-0.5	0.41	0.13	-0.22
	ENSMUSG00000007097	Atp1a2	ATPase, Na+/K+ transporting, alpha 2 polypeptide	ENSMUST00000097464	0.4	-0.32	-0.07	0.15
	ENSMUSG00000030782	Tgfb1i1	transforming growth factor beta 1 induced transcript 1	ENSMUST00000166755	0.29	-0.25	0.02	0.03
	ENSMUSG00000090733	Rps27	ribosomal protein S27	ENSMUST00000170122	0.37	-0.08	-0.02	0.31
	ENSMUSG00000032870	Smap2	small ArfGAP 2	ENSMUST00000043200	-0.62	0.37	-0.1	-0.15
